# Cybercare 2.0: meeting the challenge of the global burden of disease in 2030

**DOI:** 10.1007/s12553-016-0132-8

**Published:** 2016-05-27

**Authors:** Joseph M. Rosen, Luis Kun, Robyn E. Mosher, Elliott Grigg, Ronald C. Merrell, Christian Macedonia, Julien Klaudt-Moreau, Andrew Price-Smith, James Geiling

**Affiliations:** 1 Geisel School of Medicine, Dartmouth College, Hanover, NH USA; 2 Plastic Surgery, Dartmouth-Hitchcock Medical Center, Lebanon, NH USA; 3 Thayer School of Engineering, Dartmouth College, Hanover, NH USA; 4 Global Citizen Safety and Security WG; IEEE Society on Social Implications of Technology (SSIT), Vienna, VA USA; 5 Anesthesiology and Pain Medicine, University of Washington, Seattle Children’s Hospital, Seattle, WA USA; 6 Virginia Commonwealth University, Mentone, AL USA; 7 Lancaster General Health, Penn Medicine, Lancaster, PA USA; 8 Department of Political Science, Colorado College, Colorado Springs, CO USA; 9 Veterans Affairs Medical Center, White River Junction, VT USA

**Keywords:** Global health, Burden of disease, Cybercare, Health care cost, Telemedicine, Cell phones, Information technology, IT

## Abstract

In this paper, we propose to advance and transform today’s healthcare system using a model of networked health care called Cybercare. Cybercare means “health care in cyberspace” — for example, doctors consulting with patients via videoconferencing across a distributed network; or patients receiving care locally — in neighborhoods, “minute clinics,” and homes — using information technologies such as telemedicine, smartphones, and wearable sensors to link to tertiary medical specialists. This model contrasts with traditional health care, in which patients travel (often a great distance) to receive care from providers in a central hospital. The Cybercare model shifts health care provision from hospital to home; from specialist to generalist; and from treatment to prevention. Cybercare employs advanced technology to deliver services efficiently across the distributed network — for example, using telemedicine, wearable sensors and cell phones to link patients to specialists and upload their medical data in near-real time; using information technology (IT) to rapidly detect, track, and contain the spread of a global pandemic; or using cell phones to manage medical care in a disaster situation. Cybercare uses seven “pillars” of technology to provide medical care: genomics; telemedicine; robotics; simulation, including virtual and augmented reality; artificial intelligence (AI), including intelligent agents; the electronic medical record (EMR); and smartphones. All these technologies are evolving and blending. The technologies are integrated functionally because they underlie the Cybercare network, and/or form part of the care for patients using that distributed network. Moving health care provision to a networked, distributed model will save money, improve outcomes, facilitate access, improve security, increase patient and provider satisfaction, and may mitigate the international global burden of disease. In this paper we discuss how Cybercare is being implemented now, and envision its growth by 2030.

## Introduction

This paper proposes transformative advances to our healthcare system that might mitigate the international global burden of disease [1] and help fix the “broken” healthcare system so often decried in the United States (US) [2]. We propose to address the challenge of providing health care both internationally and domestically using a model of networked health care called Cybercare [3], originally derived from the expression “Health care in Cyberspace.” This model shifts health care from hospital to home; from specialist to generalist; and from treatment to prevention. Moving health care provision from a central, hospital-based model to a networked, distributed model will save money, improve outcomes, facilitate access, improve security, and increase patient and provider satisfaction. Cybercare employs technology to deliver services efficiently across a distributed network — for example, using telemedicine, wearable sensors, and cell phones to link patients to specialists and upload their medical data in near-real time; using IT to rapidly detect, track, and contain the spread of a global pandemic; or using cell phones to manage medical care in a disaster situation. Cybercare uses seven “pillars” of technology to transform medical care delivery: genomics; telemedicine; robotics; simulation, including virtual and augmented reality; AI; the EMR; and smartphones. All these technologies are evolving and blending. The technologies are integrated functionally because they underlie the Cybercare network, and/or form part of the care for patients using that distributed network.

**Fig. 1:**
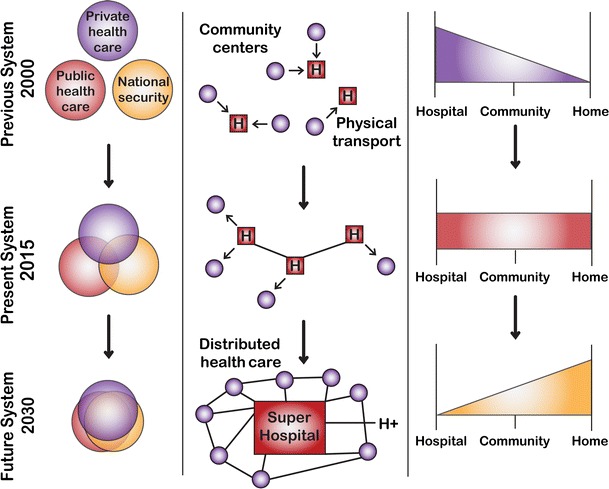
How Cybercare will make the health care system evolve over time. 1. In 2000, the bulk of health care was centered in large hospitals, to which patients were transported often over long distances and at large expense. 2. In 2015, health care has been distributed away from central hospitals, with more care provided in community clinics and at home through telemedicine and wearable sensors. The network helps to integrate the functions of private and public health care and national security (the healthcare network also functions in disasters or acts of war) 3. In 2030, Cybercare will have enabled the bulk of care provision to happen at home, with only “super hospitals” remaining for very specialized services. The functions of private and public health care and national security almost completely overlap

The Cybercare model is already being implemented in the US and worldwide; in this paper we discuss the progress of Cybercare to date and how we expect it to evolve through 2030 (see Fig. 1). Section 1 discusses current demographic and epidemiologic trends that challenge the practice of medicine worldwide, today. Section 2 describes how Cybercare could solve these current problems and develop to meet the medical needs of the world we envision in 2030.

### Providing medical care — a domestic and international crisis

The news about today’s international and domestic public health is rarely hopeful. Migrant populations, crossing natural and political borders, spread communicable disease; international air travel facilitates this process. Maternal and childhood illness remain major public health threats in the developing world. In the first world, aging populations suffer a burden of chronic non-communicable illnesses, such as vascular disease, diabetes, cancer, etc. Aside from the global burden of disease, US health care is still too expensive for many patients; physicians are pressed to increase revenue while hospitals and medical centers profit from people’s illness; insurance is a headache for patients, lawmakers and doctors, with no clear winners except insurance companies; and the focus of care remains on treatment rather than prevention. The current healthcare system, based on care provision at central, expensive hospitals, is not financially efficient, practical for providers, or accessible to all patients. Efforts to fix the “broken” system have involved cutting costs and services, limiting clinicians’ ability to practice, or giving patients too much or too little care, without attention to outcomes.

### Global burden of disease

Dr. Christopher Murray’s 20-year Global Burden of Diseases (GBD) initiative documents the incidence and prevalence of disease and injury for 188 countries between 1990 and the present [4]. GBD is a “*systematic effort to quantify the comparative magnitude of health loss due to diseases, injuries, and risk factors by age, sex, and geography over time…this pioneering effort continues to be hailed as a major landmark in public health and an important foundation for policy formulation and priority setting”* [5]. This excellent system helps us plan how medical resources should be allocated to combat disease. The GBD study classifies worldwide disease into non-communicable disease and communicable disease. At present, non-communicable diseases (such as cancer, diabetes, heart disease, etc.) are overtaking communicable diseases (such as influenza, Ebola, HIV, etc.) in numbers and costs. Furthermore, the prevalence of diseases associated with famine and malnutrition is now being surpassed by the incidence of diseases associated with being overweight. It is astounding that more people now die from obesity rather than starvation (Maureen Quigley, personal communication). These epidemiological trends correlate with demographic shifts to increase the burden of disease.

### Demographic shifts

Worldwide demographic changes drastically impact global health, and can increase the cost of healthcare delivery systems across the planet until these systems become unsustainable. The pattern of diseases is substantially different between developed and developing nations.

#### Third world: rapid population growth; migration into cities

In the third world, rapid population growth in Africa and Asia, combined with the migration of refugees into cities, results in increased cost to treat communicable diseases. According to the Population Institute [6] “*the extreme and rapid expansion of human population — now counted by an additional BILLION people every 12 to 13 years — is mortally taxing the Earth and its resources.*” These populations are centered in cities: WHO’s Global Health Observatory data website [7] states: *“The urban population in 2014 accounted for 54 % of the total global population, up from 34 % in 1960, and ⋯ by 2017, a majority of people will be living in urban areas.”* For example, the city of Lagos, Nigeria, has 21 million people [8].

#### Developed countries: graying of the population, chronic diseases of the elderly and obese

The number of adults over 65 in the US is expected to grow from about 35 million in 2000 to 71 million by 2030. In developing countries, the number of people over 65 is projected to nearly triple from 249 million to 690 million [9]. In the US, the Centers for Disease Control and Prevention (CDC) [10] estimates that chronic diseases associated with aging — such as heart disease, stroke, cancer, diabetes, obesity, and arthritis — are among the most common, costly, and preventable of all health problems (non-communicable diseases). According to a study [11], “*86 % of all health care spending in 2010 was for people with one or more chronic medical conditions*.”

The healthcare expense will increase both from population growth and ageing: there are more people being born, and more older people staying alive.

### A borderless world

The twenty-first century is a global era: populations are no longer limited by boundaries, and borders do not exist in the same way as in previous centuries. Air travel allows people and diseases to rapidly cross the globe, dramatically increasing the impact of infectious diseases. The migrant and refugee crisis in North Africa and the Middle East has forced immigrants to cross boundaries by land and sea, often at great risk to their own well-being. While populations are forced into neighboring states or countries, healthcare systems are not designed to manage a dynamic shift in population. Patients’ medical records and critical life-support medications or supplies do not transfer with them, because IT systems and policies don’t yet exist to support this process internationally. The fact that we now live in a borderless world with shifting demographics has worsened the crisis in health care and burden of disease.

### Discussion: Christopher Murray’s burden of disease study

In their influential studies, Murray and Lopez [12–14] argued that the burden of disease would shift from communicable diseases that predominantly afflict the poorer populations of the planet towards issues of chronic illness. This shift prioritized the diseases of the wealthy (obesity, smoking and lung disease, heart disease, etc.) while diminishing the emphasis placed upon basic principles of public health such as clean water, basic health infrastructure, preventive health care, or disease surveillance in the developing world.

As a result of these influential studies, international organizations such as the World Health Organization (WHO) and the Pan American Health Organization (PAHO) have directed significant international aid towards the treatment of chronic illnesses of affluence, and since 2011 funding has moderately declined for surveillance and treatment of communicable diseases of the global poor such as malaria, tuberculosis, cholera, dysentery, etc. This is true both in the US and internationally:

- As delineated within the US Health and Human Services budget, US expenditures on prevention of chronic disease reached a zenith of $1.188 billion in 2014, but then experienced a minor decline to $1.078 billion in 2015. This recent change in long-term trajectory of funding is partially attributed to the recognition that communicable disease remains a significant and persistent threat to global health and to the health of the American people, largely as a consequence of the Ebola epidemic of 2014–15, described later in this paper [15].
- The long-term decline in funding for communicable disease, and increased funding for chronic illness continues at the global level, reflected in the budget of the WHO. WHO funding for communicable diseases declined significantly from $913 million in 2012 to $841 million in 2014–15, a net reduction of $72 million. Over this same time span, funding for non-communicable diseases increased from $264 million to $318 million, an increase of 20.45 % [16].
- The WHO budget for 2016–17 projects an increase to $339 million for non-communicable disease, whereas the budget for communicable disease is expected to decrease to $765 million in 2016–17 [17].
- If we combine the data sources above, consequently (despite the considerable economic and political destabilization generated by the Ebola epidemic of 2014–15), the WHO budget for communicable illness is projected to decline significantly from 2012 to 2016, from $913 million to $765 million, a decline of 16.21 %.

Even as Murray and Lopez’s report dismissed the probability that communicable disease would continue to afflict significant proportions of the global population, these diseases have not disappeared. For example, malaria still affects a significant proportion of the global population, and this effect is concentrated within the poorer populations in the developing world. According to the WHO, 3.3 billion people are at risk of malaria infection, 1.2 billion of those are at high risk of contracting the illness, and ongoing transmission is occurring in 97 countries. In 2013, there were an estimated 198 million cases of malaria, and approximately 580,000 deaths, with 90 % of this morbidity and mortality occurring in Africa [18]. Even with the WHO trumpeting recent reductions in incidence and prevalence, one can hardly state with any degree of veracity that malaria is on the edge of eradication.

Infectious diseases still manifest in the form of deadly epidemics that sweep through impoverished populations, and through states that exhibit weak healthcare infrastructures and profound inequalities. For example, the Ebola epidemic of 2014–15 ultimately claimed circa 11,000 lives, damaged economic productivity in West Africa, and undermined effective governance in affected nations [19]. The expansion of cases of Ebola into Zaire, and beyond West Africa to the US, Spain, and United Kingdom (UK), generated a global epidemic of fear. Ultimately, the socio-political destabilization generated by the epidemic resulted in its designation as a threat to national security by the US, and to international security by the United Nations Security Council in Resolutions 2176 and 2177 [20, 21]. As a second example, the epidemic of cholera that has swept across Haiti since 2010 has caused 8768 deaths and sickened approximately 750,000 [22]. It has also undermined the legitimacy of the United Nations’ (UN) operations in Haiti, and negatively affected the perceived legitimacy of the Haitian government. The disease has spawned riots against both the government and the UN for their failure to control the spread of the bacterium.

Pathogens move about the planet courtesy of human vectors, using rapid modes of transportation such as airplanes, and they ignore borders. The borderless world has many severe consequences, including the fact that it’s very hard to prevent or contain pandemics. Perhaps the greatest failure of the GBD report is its ignorance of the evolutionary capacity of pathogens to adapt. Pathogens are not static; they exhibit rapid evolution in response to environmental conditions. As Price-Smith has argued, pathogens are constantly evolving to exploit novel ecological conditions that arise from new ecosystems, and many of these pathogens have arisen under conditions of relative prosperity. As such, these “plagues of affluence” now afflict people throughout the developed world, radiating throughout the new sanitized environments of technologically sophisticated ecosystems including hospitals and office environments [23]. Examples of pathogens that thrive in these new sanitary ecologies are Vancomycin-resistant enterococcus (VRE), and Methicillin-resistant Staphylococcus Aureus (MRSA), etc. Thus, the GBD study, which held that infectious diseases would simply be eradicated, did not account for the genetic mutability of pathogens; their ability to colonize novel ecological niches within sanitized societies; or how much they impact health and healthcare cost.

Regarding the pandemics, as an example, Cybercare can help mitigate the spread of disease and its impact: we can use advanced technology and databases to track individuals, including data on when and where the infection came from, and bring the appropriate resources to solve the problem before infection grows exponentially. Over time we might employ more advanced technology to foresee and adapt to the rapid evolution of infectious diseases. For example we could use simulation models to predict the spread of disease, based on gathered data that include population demographics, climate and environment, and response systems, both locally and globally.

This section has discussed many pressing world problems in health care today. The following sections describe Cybercare, how it is already addressing many of these problems, and how it should change from now to 2030 to address the current issues and adapt dynamically to unforeseen issues that will arise.

## Cybercare as a solution to the global burden of disease

We can respond to both the domestic and international healthcare crises described above, using Cybercare’s advanced technology to provide distributed health care. For more than 40 years researchers have written about applying computer technologies to improve medical care. During this period, government and private agencies have collected and organized vast quantities of scientific data that relate to our health. Many illnesses and injuries are directly caused or significantly influenced by our food, air, water, medications, and environment. This section of the paper will describe and show examples of how information technology, when applied to health care and public health, may help avoid or delay disease, foster prevention and wellness, improve quality of life, minimize healthcare expenses, and predict future epidemics or healthcare crises.

The current healthcare system, in the US and most of the world, is still based on a 20th century model in which patients travel, often long distances, to centralized hospitals and pay a high price for treatment. This outdated model concentrates physical resources in centralized hospitals for the convenience and efficiency of healthcare providers. For patients, this design creates access challenges and encourages detrimental cross-pollination like hospital-acquired infections or medication mix-ups. It also produces a system with large critical nodes that are vulnerable (see Fig. 1). For instance, a natural disaster or a terrorist event could take down a hospital along with its providers, causing all patients to forfeit care. This happened in Toronto, Canada, in 2003 with the severe acute respiratory syndrome (SARS) epidemic. De-centralizing hospitals and moving more sophisticated care into the community and home with Cybercare makes patients better able to access and dictate care, and makes the system more robust during normal operations and when under threat.

The following sections define Cybercare; describe its technologies and applications, and provide a case study of how Cybercare would improve care in a pandemic.

### Cybercare: definition and model overview (Elliott Grigg)

#### What

Cybercare means “health care in Cyberspace”. Cybercare provides a network-based healthcare solution that de-centralizes resources using IT to deliver health care across a network – for example, doctors consulting with patients via videoconferencing; or patients receiving care locally — in neighborhoods, “minute clinics,” and homes — using telemedicine, smartphones, and wearable sensors to link to tertiary medical specialists. This model contrasts with traditional health care, in which patients travel (often a great distance) to receive care from providers in a central hospital.

The Cybercare model is robust, efficient, and accessible. A key concept of Cybercare is that instead of patients moving (from remote areas to hospitals), information moves (from a centralized area with specialists to remote areas with generalists and patients). The site of care moves from centralized hospitals to neighborhood clinics and homes; care provision shifts from specialists to generalists; and clinicians promote prevention over treatment. We then need only a few remaining, centralized “super hospitals” to provide specialized care and information (see Fig. 1).

Cybercare has seven pillars of technology (genomics, telemedicine, robotics, simulation, artificial intelligence, the electronic medical record, and smartphones) — which constantly evolve and overlap. In the past decade, the use of genomic data to truly personalize care has become prominent. Since 2004, the US has focused on creating a complete personal health record for each citizen and developing technical standards to allow near-real-time health data acquisition from medical devices, health practitioners, caregivers, and/or patients themselves. Simulation technology combined with telemedicine empowers the generalist to provide care at the level of the specialist. EMRs, cell phones, and sensors empower the patient provider to care for him- or herself with the help of a nurse practitioner (NP), physician assistant (PA), or family member in their home or local clinic. Artificial intelligence can be designed into laboratory tests that tell the doctor when the test is complete, through the use of intelligent agents assigned to a laboratory test or imaging study. Smart robots exist that could help the patient conduct or interpret lab tests and other tasks in the home [24], and many more are in development. Emphasis is placed on the prevention of disease and living a healthy lifestyle. The network provides information and tracking that enables better prediction of disease pandemics, so that clinicians can intervene earlier. Patients can be treated remotely during disasters, when they are mobile, or traveling. This new web of care replaces the present system of centralized hospitals that is inefficient, expensive, and forces the patient to travel rather than receiving care in their location.

#### How

The Internet, mobile computing, and inexpensive sensors offer an opportunity for us to democratize health care and make the overall system resilient. Today most people in every country carry computers in their pockets (i.e., smartphones) that are several-fold more powerful than the ones used to send the Apollo missions to the moon. Each phone is wirelessly connected to the Internet; most are sensor-laden and able to record health and activity metrics.

**Fig. 2:**
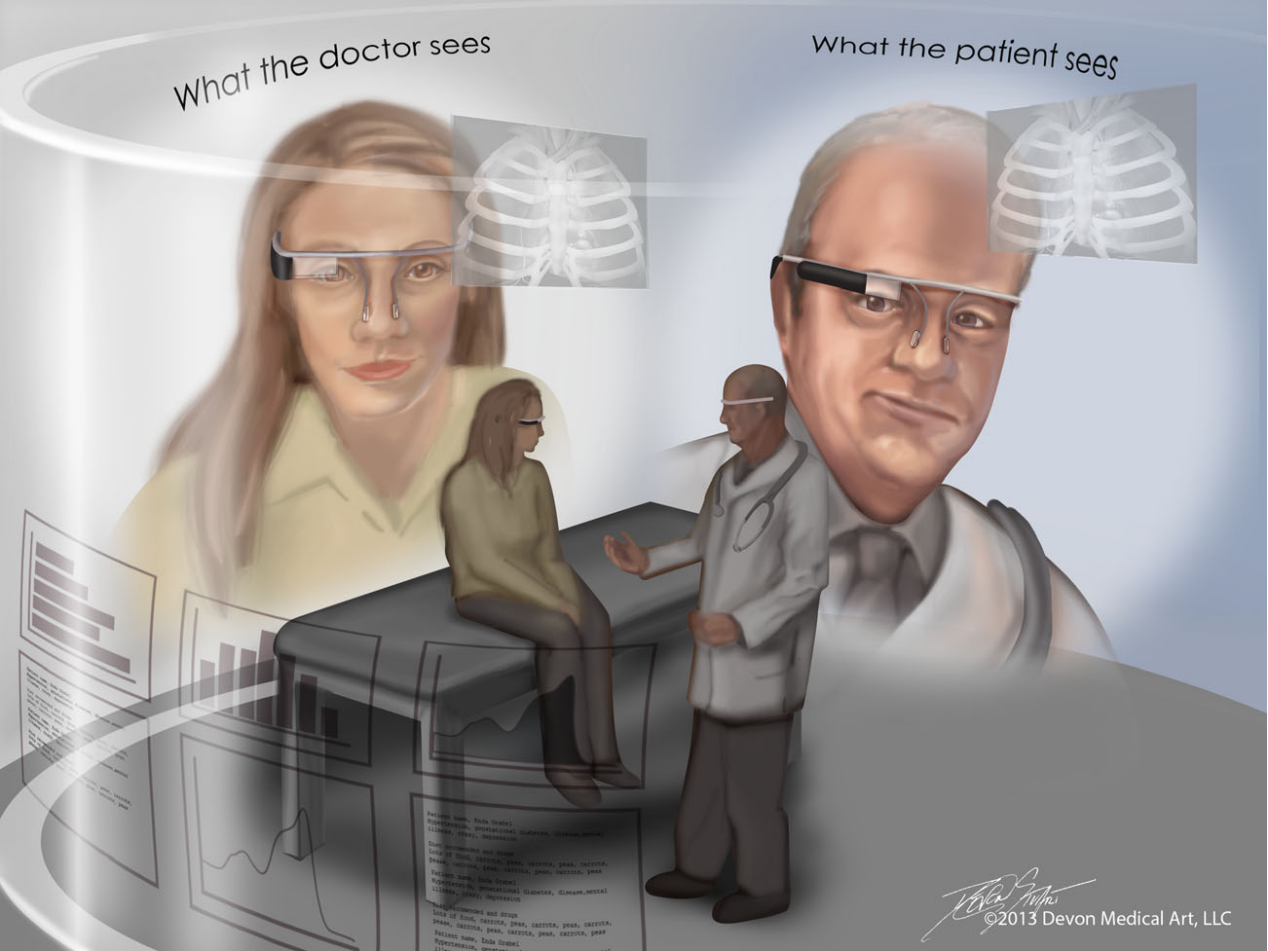
Augmented Reality. What the patient and the doctor would normally see in their field of view is augmented with extra data — in this case imaging scans and chart notes — that help the provider to better diagnose, discuss, and communicate medical information with the patient

Procedures are becoming less invasive, and complex surgeries are performed intravascularly or endoscopically. Sophisticated robots such as daVinci allow surgeons to perform elaborate operations remotely. Robots can enhance human performance and increase the level of safety in surgery. Simulation trains generalists to provide higher-level specialty care, and augmented reality allows them to use real-time information to provide specialized care (see Fig. 2). This also allows information like genomics to be applied when seeing the patient. Big data can be made available for the generalist or the patient to use in caring. Simulation models can also predict outcomes for patients based on their daily activities, risk factors, and biomarkers from their genomics.

Laboratory tests and other inexpensive, portable, efficient point-of-care technologies can connect to the cloud for analytical tools. This has been done in Indonesia, Singapore, and Australia by Oracle using Health Connect ™ software [25, 26]. Biotechnology produces cures for ailments by pill rather than a morbid surgery. In essence, many pharmaceuticals are performing ‘surgery’ at the microscopic, cellular level. Three-dimensional printers — currently in their infancy — portend a future in which individuals could compound prescriptions in the home using basic ingredients. Many consumer services may soon deliver goods and services, within hours of online ordering, and someday by drone. This is being done in the retail industry now for consumer goods.

#### Impact

When patients have easier access to and control over the generation of their health information, the patient-physician dynamic will change. Patients will become more like customers (and less like passive patients), and providers will become more like consultants (and less like clergymen). Control over their health information will dramatically empower patients and will likely force the healthcare industry to perform more like traditional service industries where customers dictate much more of the interactions.

**Fig. 3:**
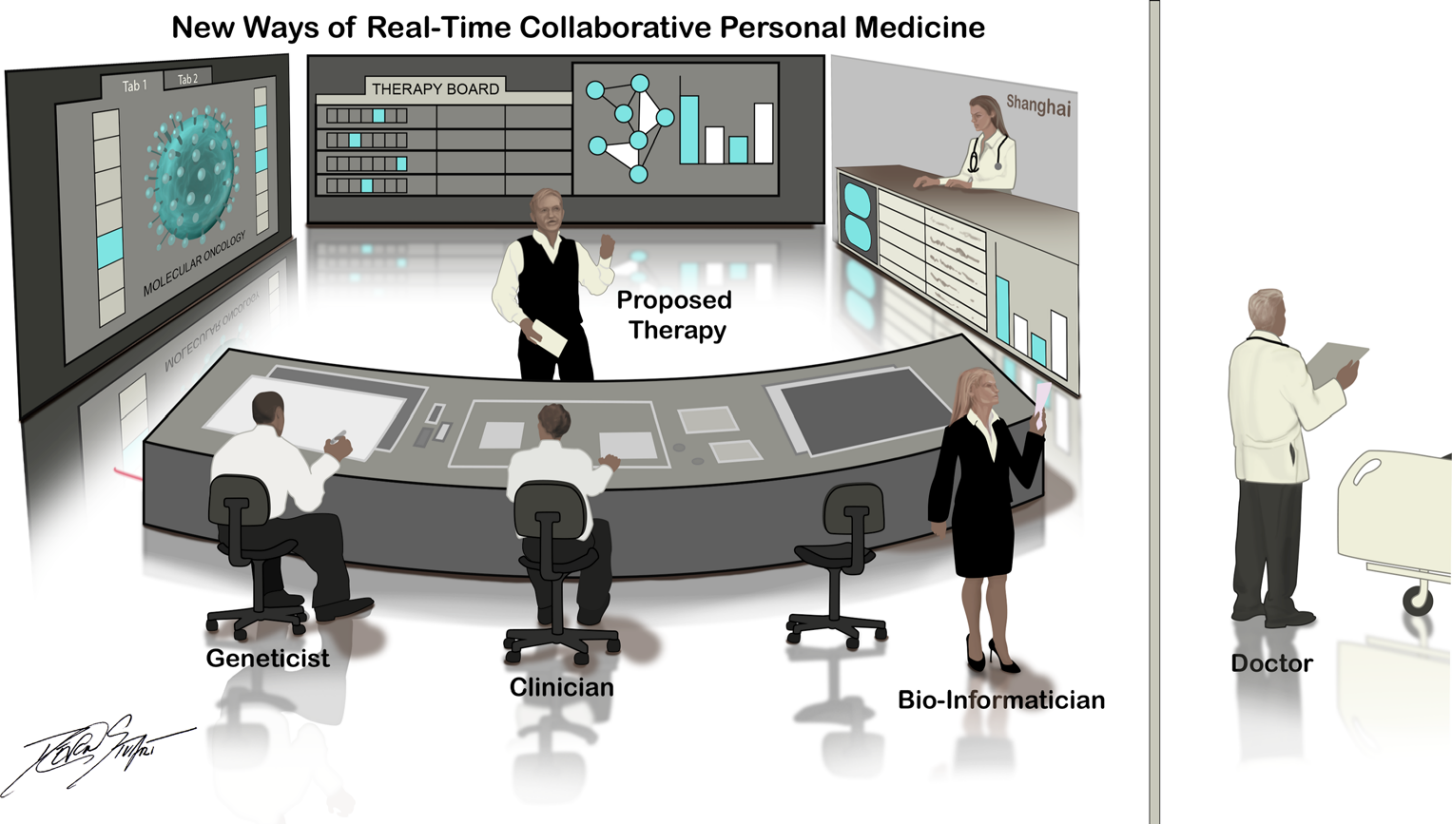
Collaborative Personal Medicine. A room in which all data about a patient is being integrated to make personal individualized medicine. This data is then presented to the patient’s physician and in some cases also to the patient directly

Remote monitoring will enable more preventive medicine, as providers are given access to continuous data streams from the home. When these data streams are combined, much larger and more compelling outcomes studies — with enrollment of millions rather than thousands — will answer previously unanswerable questions about the effectiveness of interventions. Lessons from these large data sets will in turn be applied to individuals to better customize therapies based on genetic or demographic idiosyncrasies (see Fig. 3).

When health care is more ubiquitous and mobile it will also be more accessible to rural and remote locations via telemedicine. Remote consultations will evolve into remote interventions, saving patients the cost and complexity of transportation. When health care is more accessible it will be more utilized and we will prevent more ailments from progressing. Health care will shift from an emphasis on treatment to an emphasis on prevention by promoting more healthy lifestyles with exercise and healthy diet.

Finally, a more robust and distributed system with fewer critical nodes will be more resilient to threats both natural and man-made. Under Cybercare, the loss of a single facility (like a trauma center) will not cripple the system in the same way as it would today. And the ability of medical systems to scale to a natural or biological disaster will be enhanced greatly as the load can be distributed more evenly across the system (see Fig. 1).

#### Progress of Cybercare to date: virtual medicine

Health care has progressed from 2000 to 2015 as care has become more decentralized, in many cases moving from hospital to clinic or to the home. Point-of-care devices have converted the former doctor’s house call to a virtual call with telemedicine and diagnostic, inexpensive devices that are wearable and available to the patient. With today’s technology we could even soon place an inexpensive robot in the home — a “healthbot” to provide telemedicine and some basic hands-on skills, like taking a blood pressure or dispensing pills. Medical robots currently in use or development include swallowable capsule robots (that can carry cameras to observe and diagnose; tools to take biopsies; sensors to check tissue; and needles to administer drugs), therapy robots, exoskeletons, and more [27]. Virtual medicine will provide care in the patient’s own environment through the introduction of virtual technologies like telemedicine.

Cybercare can provide a virtual environment in which to care for a patient with morbid obesity and all of its secondary consequences. Cybercare can educate this patient in what to expect if the weight is not controlled; that the patient may develop diabetes type II or early arthritis. The environment can include online patient support groups in which patients help each other to control their diets and to exercise. It can connect with their “fitbit” or smart device to control their weight through a calculated exercise and diet program. Cybercare can connect the patient to their provider whether this is a primary care physician (PCP), NP, or PA. This will be an integrative approach: all of this is part of the present and future virtual healthcare medical environment.

As we look toward 2030, we expect the hospital’s central role to diminish. Many fewer hospitals will be required, and those remaining will run in a cost-effective and efficient manner (see Fig. 1). Like factories, hospitals will be run 24/7 as a limited, expensive resource. This is already happening as radiology runs magnetic resonance imaging (MRI) and computerized tomography (CT) machines on two or three shifts, the emergency room runs 24 / 7, and some hospital clinics such as dermatology and pediatrics are open nights and weekends. Robotic surgery equipment is expensive, and should be utilized around the clock. The former hospitals’ role in providing intermediate and basic care will transition to smaller clinics in neighborhoods, drugstores, and shopping malls; some care will be provided in the home. Even the house call will return, but through telemedicine links to the home from the doctors’ office in the clinic or hospital. This will spare patients the expense, hassle and health hazards of being moved to a hospital where they are at a greater risk of nosocomial, hospital-acquired illnesses.

The few remaining hospitals will be specialized “super hospitals” for care that still cannot be provided in a distributed manner (see Virtual Valley Forge section). An example would be face transplants that require large coordinated teams of specialists. Cybercare will be used every day to deliver care to the common problems like obesity and the common cold, and it will be available for both natural and man-made disasters. We will see this transition over the next 15 years.

The Cybercare healthcare model is a dual-use system. It is used every day for health care prevention, diagnosis, and treatment. It will also be available for disaster whether natural, man-made, or intentional (terrorism). As seen by the recent attacks in Paris, Beirut, Bamako, and San Bernardino, California, we need to have a healthcare system that can respond to whatever needs are presented.

#### Issues

One of the problems in decentralization is a lack of enough providers, including surgeons, as discussed in the Lancet Report 2030 [28] and by the Association of American Medical Colleges [29]. Cybercare would handle this problem through task shifting. Through training, simulation and augmented reality, generalist medical doctors (MDs) can perform some specialist tasks, and PAs and nurses can perform some generalist MD tasks. Specialist consultations will be done via telemedicine, and other simpler tasks by robots, or by the patient as he or she reads data from body-worn sensors. Family and community members could receive training on first aid and basic emergency medical procedures.

Many of the existing barriers to this distributed, networked healthcare model are more regulatory and legal than technical. The fragmented electronic health record landscape and the lack of interstate health delivery regulations (in the US, or similar standardized laws between countries) are the only things standing in the way of many of these changes. Much of the available information across multi-disciplines and inter-disciplines today exists in silos, and thus, is not used or shared as it could and should be. As the technology evolves, the regulatory landscape will have to evolve to better accommodate the reimbursement and liability of telemedicine, and to address other interoperability issues.

### Genomics: the patient provider

**Fig. 4:**
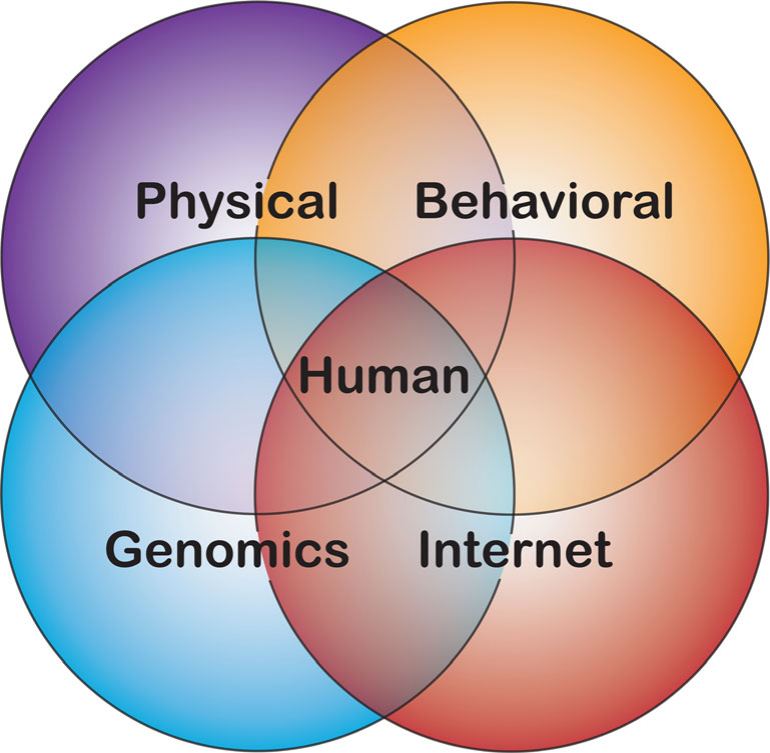
Venn Diagram. Cybercare will help the provider to view all data from a patient’s physical, behavioral and genomic traits, through the integration of the Internet and its information technology tools

Just as Cybercare shifts care from the hospital to the home, it can also shift care from the provider to the patient. One immediate goal of Cybercare is to address the shortcomings of our current system of health care, which can be facilitated by empowering the patient to take part in providing his or her own care. The family members will also take on an expanded role in the care of the patient. This would be known as the patient provider model of care. Previously, health care was dominated by the patient-provider relationship wherein the patient passively looked to a provider for direction regarding his or her health. We believe that Cybercare will augment this relationship by integrating genomic medicine, wearable technology and preventative medicine. This gives the patient provider an in-depth look at the patient’s physical traits, behavior, and genomics through the integration of the Internet and its information technology tools (see Fig. 4).

The Human Genome Project (HGP), a substantial international initiative, was tasked with identifying the sequence of base pairs within human deoxyribonucleic acid (DNA). Originally, scientists had predicted that the HGP would collate a large number of genomic sequences into one uniform database. Physicians would use this information coupled with their previous expertise to provide personalized care for their patients. Until recently, genomic sequencing was limited to a rather specific patient population, as the cost far outweighed the benefits for most patients. That has now changed with Illumina claiming to have the ability to sequence the genome for $1000 and the overall decrease in cost seen since the early 2000s [30, 31]. Due to the falling cost of sequencing and the proliferation of direct-to-consumer tests, such as 23andme.com and ancestry.com, we predict a greater shift towards genomic-based medicine. In order for the patient provider to adequately utilize this information they will need to track their day-to-day activities using wearable technology. This is already being done for small genomes like viruses. By 2030, it should be possible to use a smartphone to do genome analysis.

We are in the midst of a mobile revolution wherein patients can individually track their vital signs, measure and record caloric intake, and provide advice based on that data. This data can then be used to predict health outcomes over time and predict trends of care. As a patient provider, each patient would be responsible for compiling his or her personal chart by utilizing wearable technology, which includes smartphones, tablets, and smart watches. The expectation would be that the patient and the provider act as a team to develop the best treatment possible. In the case of obesity, this could mean increasing daily fitness, decreasing caloric intake, or genetically modifying pre-existing DNA. Technology will play a vital role in enabling the patient to be a co-provider. By the year 2030, we expect mobile technology coupled with genomic information to shift the responsibility of care from the physician to the patient. Furthermore, we expect a major shift from treatment-based care to preventative care, with a subsequent dramatic reduction in the overall cost of health care.

The goal of Cybercare is to efficiently allocate resources to prevent disease before it ever materializes in the patient. The notion that we can prevent major complications from ever occurring rather than treating them is not new, yet we have failed to integrate preventative medicine with modern medicine. For example, obesity is a rising issue worldwide according to the GBD study, and we could lessen its impact by better educating the patient through frequent reminders on personal devices (such as a reminder to exercise or adjust diet according to daily intake) and the device transmitting patient performance data back to their provider. As of 2008, there were 1.48 billion overweight adults worldwide [1, 32]. The Cybercare goal is to shift focus from response-driven procedures such as bypass surgery towards healthy living. To achieve this goal of preventive care, the patient population will need to take on greater responsibility regarding their own health. The system in which patients are passive about their care is no longer sufficient and the patient should dictate the direction of his or her own care as a co-provider.

By integrating the previously mentioned components into a cohesive model of care, there is a distinct possibility that Cybercare could increase our nation’s health, as measured by disability-adjusted life years (DALYs) [1]. DALYs take under consideration not just the age at which one dies but also the quality of life leading up to an individual’s death. When discussing treatment vs. prevention it is critical that we mention the quality of life of our patients. If we don’t improve the length and quality of life for our patients, then we are not providing an innovative enough solution to the current healthcare crisis.

### Telemedicine

Telecommunications and IT have been applied to support the delivery of health care for more than 20 years. The term telemedicine has been generally applied to this activity and may be considered synonymous with e-Health, Telehealth, Telecare, etc. Telemedicine has generally been seen as connecting a patient to a provider or a provider to another provider/specialist. When telemedicine is on a network then it comes under the umbrella of Cyberhealth/Cybercare.

In 2009 the United States implemented the Health Information Technology for Economic and Clinical Health (HITECH) Act, which required that an electronic health record would be the foundation for healthcare delivery in the US. The law laid out five meaningful uses of an electronic record: quality, engagement of patients and families, improved care coordination, improved population health and public health, and privacy protection. Making health care digital and interoperable rapidly made distance irrelevant to practice. The act is currently in its second iteration of requirements and in 2015 is challenged to demonstrate improved outcomes [33]. The US Affordable Care Act (ACA) of 2010 offers hope for near universal coverage and the emphasis on patient rights, access, affordability, and a coherent funding plan has seen millions of new insurance beneficiaries [34]. If the HITECH initiative made the EMR a prime tool for telemedicine, the ACA created a surge in demand for health care that exceeds capacity. The American Association of Medical Colleges predicts that by 2025, the US will have a shortfall of 25,000 to 50,000 physicians [29]. A shortfall of surgeons is also predicted by the Lancet 2030 article [35]. Task shifting can help to reduce this shortage of physicians by utilizing simulation and augmented reality technologies under Cybercare. The situation in nursing is even more dire. Instead of relying on nurses, a patient’s family members, with proper training, could provide the necessary care. That implies no change in the established, long-revered practice pattern (called incident care) whereby a patient recognizes a problem, seeks a medical encounter, gets examined, and treated. Unfortunately we cannot staff medicine in the same way for what now is needed: the long-term management of chronic disease in an aging population. Fortunately, telemedicine’s efficiency is essential in a new care pattern that will involve much work redesign and exquisite technology [29]. Telemedicine as a tool can provide affordable and accountable access.

Over the course of the last two decades the American Telemedicine Association (ATA) and the International Society for Telemedicine and e-Health (ISfTeH) have promulgated energetically for telemedicine, patients, practitioners, and evidence-based technology. The ATA has extensive programs for research, education, and training. The ATA accredits not only educational programs but very advanced notions of direct patient contact with telemedicine services [36]. The ISfTeH is in discussions with the WHO to promote international access to health care through telemedicine, and has chapters in 90 nations/territories with established strategic plans for telemedicine [37]. The preparation of health workers in the use of electronic communication and records is best described as the empowerment of information managers who can collaborate and consistently apply evidence-based medicine (as shown in Fig. 4). This model applies equally in medicine, nursing, pharmacy, rehabilitation, and among first responders.

The use of telemedicine as a tool has led to demonstrable benefits in outcomes in chronic disease management [38], ICU [39], ophthalmology, dermatology, psychiatry, pediatrics, correctional facilities, remote sites, home health care, nursing homes, and face-to-face encounters between providers to better coordinate care. Emergency medicine, pathology, and radiology are certainly powerful performers in the steadily expanding scope of telemedicine. In well-prepared programs, the quality of outcomes is comparable to that of traditional face-to-face encounters and the cost is lower. Telemedicine is widely applied by the military, disaster management, and non-governmental organizations (NGOs) in world relief. Telemedicine has consistently shown the validation of technology that can accurately capture physiologic parameters wirelessly and with integrated transmission. Cybercare is the use of this telemedicine system over a network of patients and providers.

The quality of imaging has steadily improved with standards for radiology (DICOM) and even cell phone images to bring true representation to distant sites. The operating room has been opened to distant collaboration by high-quality video transmission. Having virtual medical staffs, i.e., physicians and knowledge available through the network, to support care at the primary level to the patient and generalist, has steadily enhanced the efficiency of medical staffing. A panoply of medical skills can be brought to bear at a distant site not by the dispatch of the expert but by linking the patient site with rare specialists in major centers. For example, a dermatologist at a remote site can view a video of the patient’s skin and talk to the primary care provider and patient about it on live video. Specialty clinics in dermatology can run a full schedule of consults by store-and-forward technology and direct patient interviews to manage difficult problems with accuracy and unprecedented access. The same can be said of retinoscopy in diabetes, tough pathology calls, and the intimate management of difficult ICU cases. Improved management of pregnancy and childhood development should come from constant surveillance, patient empowerment, and data control.

In the future, super hospitals will have limited resources of super specialists available in the rare cases that they are needed anywhere in the network. The super hospital’s virtual medical staff can become tightly integrated through professional communication and programs of quality assurance. This reduces costs: the system avoids duplication testing, reduces emergency and hospital visits, avoids transfers to other facilities, and improves disease management.

Telemedicine has been widely accepted internationally where available; there is considerable interest from patients who travel and want to access quality medical care while away from home, which has consistently led to high patient acceptance. The technology of transmission has expanded greatly with wireless telemetry, the near ubiquity of the Internet, cellular telephony, and the marvel of electronic records. Cell phones now have thousands of apps for medical support and practice, and full incorporation of social media into medical and public practice is upon us.

The overwhelming evidence for telemedicine has had a significant impact on legislation and financing. Twenty-four US states have mandates that insurers cover telemedicine consultation. Medicaid in 48 states covers telemedicine visits and Medicare pays for visits in rural areas of the US. An article in Time magazine in November 2015 brought forward numerous facts about digital medicine. United Healthcare has begun covering telemedicine and by 2019 there may be 124 million doctor-patient video consults; 2015 recorded 7.2 million. Cybermedicine might save US employers $6 billion per year in coverage for their employees and 81 % of US employers are considering coverage by 2019. Consultation in primary care may take 20 days to get an appointment in the US, and costs $150, while direct telemedicine visits are usually available in a few hours and cost less than $50 [40]. Telemedicine provides the infrastructure for Cybercare in the US; and its use is growing globally, as international travel has caused people to be more dependent on telemedicine.

The scope of telemedicine must have limits but few have been documented. Even surgical procedures can be performed or facilitated by robotic means using telemedicine. Many specialty applications have been extensively explored and validated. In fact most publications in telemedicine are now reported in specialty journals as part of that discipline’s mainstream practice improvement. Telemedicine has great overlap with informatics and social planning with greater analysis of dependable aggregated data for large insurers, health systems, and ministries of health. The scope of practice is constantly under scrutiny. The expansion of telemedicine beyond simple medical conditions to complex medical management without full inclusion of at least a virtual medical team deservedly attracts the concern of medical practitioners. Indeed equivalence is the standard, lest we ever embark on the slippery slope of reduced quality in the interest of saving money. It is also a matter of genuine and reasoned concern that the long tradition of patient confidence might get lost in a rush to digital efficiency. There is no reason to expect that all concerns cannot be satisfied in the dialogue between advocates for telemedicine and their founding advocacy for patient rights and interests.

Telemedicine is not a specialty in and of itself. There is no certificate in any specialty for telemedicine as an added competence. The practice requires appropriate skills in medicine, digital tools, nursing, pharmacy, IT, and administration. Telemedicine is a tool but no longer a peripheral tool in the armamentarium of medicine. Telemedicine is part of the foundation of medicine and Cybercare. It is so thoroughly inculcated into the fabric of health care that it will move forward in the general effort to make care accessible, accountable, equitable, and affordable while maintaining the trust of the patient. What might we expect in the coming decade?

First, a note that telemedicine is a critical component of Cybercare — but Cybercare is a network system, and telemedicine often refers to site-to-site communications rather than a network. However, this could change over time.

We should anticipate great improvements in sensing with chemical analysis for such parameters as glucose and other substrates and metabolites. We should expect closed-loop management of diabetes and software-generated immediate response for drugs in cardiac conditions and pulmonary management. The technology is just so close. For immediate response, we should expect data analysis at the patient, rather than from a response center, to deliver warnings, drugs, and other interventions. The sensor technology will surely move forward with implantable devices offering greater security, accuracy, and reliability. The problems of battery life and energy requirements should make great strides through better battery technology and the use of innate body circuits to power the gathering of data and acting upon those data. Signal transfer to a local computer and beyond will improve. Furthermore refinement of cell phone apps integrated with body implementation will make the delivery of immediate information to patients and response interactions far more effective.

The gathering of data at management centers will allow longitudinal consideration of chronic conditions in ways that are now only foreseen. Better management protocols predicated on personal patterns should become the norm. Furthermore personal management of conditions based upon genomics and proteomics are certain to dominate over generalities of retrospective review. One should expect the large databases to guide epidemiology of infectious diseases, injury, violence and environmental toxicity. This technology can be misapplied as can any other without clear training, standards and full acceptance of evidence-based medicine. Progress will be marked by the accrual of the evidence to guide the proper and effective best practices.

Telemedicine will advance in the care and prevention of human disease with the rest of medicine. In the foreseeable future it is hard to imagine that the integration of telemedicine into health care will ever be an impediment to progress. The technology and practice standards of telemedicine will move forward and might even take the lead in the constant drive to enhance the human estate. In fact telemedicine has been crucial to the management of human health off the planet in the space programs. Telemedicine can embrace healthcare needs in an electronic continuum of global concern regardless of place, on or off our planet. Telemedicine is an integral part of Cybercare and virtual medicine.

### Information technology

#### IT and critical infrastructures

IT has become essential to virtually all of the world’s basic activities including education, health care, transportation, energy, food and water supplies, commerce and defense, public health, the emergency services, the financial system, etc. IT is a fundamental technology of Cybercare. In 2015 the IT infrastructure can be conceptualized as five major components, which all impact health care:

- The Internet,
- The telecommunications infrastructure,
- Wearable components (e.g., sensors, bands/watches, cell phones, glasses, etc.),
- Embedded/real-time computing (e.g., for monitoring blood pressure of a patient in a hospital or at home, or remote control of the patient’s blood pressure, and
- Dedicated computing devices (e.g., desktop computers) connected via networks

Governments should define policies so that all citizens can afford access to IT so they can equally access all the Cybercare network features. Cybercare employs IT for planning, implementing, training, and working in a homecare environment (i.e., telehealth [41] for elder citizens with chronic conditions [42]); for patients to use health-related social media, websites, blogs, and phone apps for education; and for wearable devices that provide health information to the user or her providers.

Cybercare and IT can also play a major role in the prevention, detection, and mitigation of disease. In public health, IT helps identify important patterns of behavior when used for disease prevention through syndromic biosurveillance for early warning and disease prevention [43]. Monitoring the health and wellness of a community through real-time data sets (such as their patterns of buying medical products) is a form of protection. The data sets come from sales of certain products in supermarkets and pharmacies (i.e., flu medicines for flu symptoms, anti-gas products for gastrointestinal issues) that are plotted in a Geographical Information System (see Fig. 3). Absenteeism of students from both private and public schools is then added into this plot to confirm that something (perhaps an outbreak of flu) is happening and where. It is important to note that in this case of syndromic surveillance, none of this data exists in the hospital or an emergency room since most people don’t go to either place when they only have initial symptoms. Instead they purchase certain products depending on their symptoms. In addition to prevention and detection, IT may also enable rapid and accurate identification of the nature of an outbreak and aid in responding more quickly.

#### Lack of IT interoperability, a multistate outbreak case study, and Cybercare policy

In the global economy food, drugs, and vaccines need to be traceable so that damage can be minimized and/or stopped [44]. Cybercare could have helped prevent the following problem from happening in the US.

In October 2012, the US CDC, in collaboration with state and local health departments and the Food and Drug Administration (FDA), determined (after a one-month investigation) that a multistate outbreak of fungal meningitis had occurred among patients who received contaminated preservative-free medroxyprogesterone acetate (MPA) steroid injections from the New England Compounding Center in Framingham, Massachusetts. The CDC published monthly counts of the total number of cases and deaths (see Table 1).

**Table 1:** Data for this Meningitis Multi-State Outbreak table was obtained from the CDC web site. Since its discovery on October 6, 2012, the CDC reported the number of cases monthly, identifying the states involved and the total number of deaths across the US

Date	Total number of cases	Total number of deaths
October 6, 2012	64	7
November 1, 2012	386	28
December 3, 2012	541	36
December 28, 2012	656	39
January 7, 2013	664	40
February 11, 2013	704	46
March 4, 2013	720	48
April 8, 2013	733	53
June 3, 2013	745	58
September 25, 2013	750	64

Created by Luis Kun - CHDS/NDU - April 2013, updated June & September 2013

In the US, every patient has an identification number and so does the provider. When the healthcare provider writes an order, the pharmacy creates a third number which includes the name of the patient, the physician, and the drug sold with traceability information (i.e., date of purchase, manufacturer’s name, date of preparation and of expiration, etc.).

Given that this information was available, we can ask: Why didn’t the pharmacies alert the patients (after death number seven in October 2012) that bought those contaminated injections? How can it be justified that deaths number eight through 64 occurred a year later? More important — is there a policy in place where a pharmacy needs to notify the patient either to seek attention in a medical facility or see his or her own physician? And if not, shouldn’t that be part of the way government protects its citizens? This action should be part of Cybercare policy.

Intelligent agents might be assigned to do this under Cybercare. Each patient would have an intelligent agent (a form of artificial intelligence embedded in a medical device so it can “think” and adapt and communicate) assigned to notify the system that there is a problem, and to tell the patient not to take the medication causing the problem. Intelligent agents can inhabit the network for Cybercare and run on the Internet.

#### The need for a global (technology) policy

In the 20th century, access to clean water, food and medications were taken for granted in developed nations. In the 21st century, our dependency on IT is such that practically no matter where one lives, every country and every function society does is driven by IT. All of a nation’s critical infrastructures are not only inter-dependent among themselves but with IT. Under Cybercare, IT is connected to every human — it connects the genomics, physical person, and the patient’s behavior.

By 2030, developed countries will have more elderly living with expensive, chronic non-communicable diseases, yet in Africa and Asia, about 78 % of the world population will suffer from a lack of food, clean water and energy, and communicable diseases. Most nations will be looking at a badly needed healthcare transformation where the focus will be wellness and where the main objectives are: improving quality of life, lowering the costs, and making it affordable to all. Nations with large numbers of elderly citizens (mainly developed nations) will benefit from homecare, telemedicine, and fast access to Internet. Nations that will be overpopulated (mainly developing nations) will greatly benefit from very fast Internet access to alert their citizens to disease outbreaks.

### The super hospital as the cornerstone of Cybercare: Virtual Valley Forge

To recap, Cybercare moves medical care delivery from the hospital to the community or home. However, we need to close the circle: some medical care must always be done in a centralized hospital; we certainly can’t perform a face transplant in the home. The Cybercare model includes super hospitals that provide specialized teams and environments to conduct procedures that are not appropriate for home or clinic settings. Examples include most transplants (heart, kidney, face, hand); neurosurgery; robotic/stereotactic surgery; and stem cell transplants in oncology. The super hospital is the central point of a Cybercare network. In this section, we describe a historical example of the super hospital and how its power can influence us as we look forward to the modern super hospital.

#### Virtual Valley Forge and cybernetics

When Norber Weiner first coined the term “Cybernetics” in 1948 he created the word from an ancient Greek military term “Kybernetes,” the title of the officer who ran or governed a fighting ship for the captain. No doubt Weiner used this military analogy because of the tremendous image of an individual who did not simply bark out orders (there was another officer who did that) but someone so aware of every operation of the ship as to make it sail and fight as though it were a living creature. Anyone who has seen an actual reconstruction of a Greek Trireme with a crew of 200 under sail knows what a powerful visual representation of Cybernetics Weiner was trying to evoke.

So too are we trying to use another military analogy to provide our peers with an understanding of “Cybercare” in action with the concept of a “Virtual Valley Forge Hospital.” Valley Forge General Hospital was opened around the time that Weiner first wrote about Cybernetics. It was a military hospital constructed during World War II, but operated under a uniquely modern model of how health care should be synchronized. Valley Forge was a super hospital that provided the highest level of care possible (tertiary care and above), including specialized plastic surgery, eye surgery, and other specialties for the entire nation. Valley Forge surgeons conceived of procedures never before performed, like transplants of tissues — which were performed 10 years later (the first kidney transplant by Joe Murray).

The greatest unmet challenge of that era was not how to provide care for a burn or a traumatic brain injury (TBI) or a fracture, but rather how to care for such injuries over a prolonged period of time, and with the whole individual in mind. No hospital on the planet did that level of care at the time. Many revolutionary medical care concepts came from the institution and at least one Nobel Prize in medicine emerged from there.

Valley Forge provided coordinated and holistic care at a time when American medicine was siloed and divided along disciplines. The greatest flaw in the Valley Forge model of care and ultimately the reason it eventually was closed had to do with the very practical problem of geography. In order for a wounded soldier or marine to receive care at Valley Forge they had to move to Valley Forge, sometimes for years. This flaw in the original Valley Forge is where the opportunity awaits for a Virtual Valley Forge.

Many, indeed most, medical conditions are multifactorial and involve many organ systems and combinations of issues related to the brain, the body, and the social context. With the emergence of the power of social media, mobile devices and an interconnected cloud, there is no technological impediment to distributing coordinated care wherever and whenever it is needed. Because the care provided to war veterans is not bounded by the interstate commerce clause of the constitution it can be distributed, virtually, across many states and territories.

Valley Forge General Hospital revolutionized burn care and neuro-rehabilitation, it gave birth to transplantation science, and it was one of the first facilities to integrate behavioral health with medical and surgical specialty care. These were born out of the very real needs of combat veterans of the era. So too our veterans need a medical system that works for them and addresses their needs, including the ability to access preventative as well as treatment services leveraging the advantages of mobile devices and the cloud, as well as big data analytics and artificial intelligence. They need Cybercare, which is nothing more than a virtual version of Valley Forge (see Fig. 1).

### Case study and discussion: Ebola Virus Disease 2014

To better illustrate how Cybercare works, we offer a case study of a medical pandemic (Ebola Virus Disease (EVD) spreading globally in 2014) in which patients crossed boundaries, and many providers failed to treat or contain the problem. We then discuss how Cybercare might have handled the problem. Understanding the potential response of Cybercare helps us understand this healthcare model and how it might advance between now and 2030.

#### Surveillance and contact tracing

As EVD began to spread across the affected West Africa region in 2014, identifying cases and then tracking them became paramount in stemming the tide of the infection. In contrast to many disasters, which are bounded, pandemics based on infections fail to recognize international borders or other normal boundaries. In order to contain this disease as it spread, we would have required scalable and sustained responses. These include:

1. Early recognition, coordination, and collaboration among affected nations and regions;
2. Understanding the disease penetration and transmission dynamics with surveillance and contact tracing;
3. Utilization of existing technologies in information processing and communication (such as mobile phones) to aid in better understanding the tempo and spread of the disease.

These systems, coupled with research activities, early diagnostics, tracking and mapping capabilities (especially in a mobile population), risk factor assessment and treatment effectiveness, become essential to decision-makers in implementing effective control and treatment measures [45].

Given the penetration of mobile phones in Africa, individuals seeking information about the disease, including where to refer themselves or family members for care, could provide important information regarding the potential spread of the illness. Geographic location of callers is often mandated to be available to emergency services in times of crisis. However, in humanitarian context situations, such processes may not have the regulatory precedent to be implemented, potentially hindering the response effort. During the EVD outbreak, Sierra Leone deployed caller location services within its 117 Ebola Response Centers. Two projects were implemented concurrently:

1. Cell tower locations were supplemented by information collected by 117 call operators, and
2. Real-time location services of callers were deployed rapidly to support emergency services’ response efforts.

Privacy issues did occur, though these were in part addressed with software solutions [46].

Typically, once a potential contact with a patient occurs, tracking them is a paper-based system involving data collection forms, data aggregation from local sites, data entry into a database, data aggregation on a regional scale, data review and reporting, and finally report submission to national decision-makers. Such a process can be especially challenging not only within the context of the area from which the epidemic surged, but also given the 21-day incubation period of EVD.

In 2014, a team designed and implemented a smartphone-based contact tracing system that was linked to data analysis and visualization. The project, started in Conakry, Guinea, eventually expanded into five prefecture regions over six months, tracking more than 9000 individuals. The system was based upon the CommCare mobile application and was integrated with Tableau, a business intelligence software using protocols publically available from the CDC as well as the WHO. The contact software was designed to not only intake information on affected persons, but also to track their movements using time stamps and data location. Dashboards helped to display the information and performance of the collection methodology. Data validation occurred with test comparisons with paper-based systems, eventually approaching 90 % agreement [47].

#### Education and information dissemination

Health information technology (HIT) using electronic health records (EHR) has developed with mostly passive utilization for providers to get real-time information on medications, laboratory and imaging results, and to provide a method of documenting care. Its use in emerging illnesses or disasters such at EVD is less well described. Once available, providers did embrace HIT in caring for patients suffering from the EVD disease. The WHO and the CDC actively disseminated current information on diagnosis, treatment and supportive care, such as the proper use of personal protective equipment. However, while EHRs helped support individual episodes of care, they proved less helpful in sharing that information during the outbreak. One problem that occurred was the concept of “technology-induced error” where critical data that may have proved useful in tracking the disease or evaluating it on a population basis was hindered by the non-standard placement of the information in the EHR [48].

Symptom monitoring apps and other mobile applications were less well developed during EVD, with the exception of outbreak tracking maps. Ushahidi did develop a mapping tool to track the disease using crowd-sourced data. Also, the International Red Cross sent two million text messages each month in an effort to spread current knowledge [49]. One challenge in the development and usage of such tools, however, stems from mobile phone penetration in Africa. While mobile phone use is extensive throughout much of Africa, smartphone availability is less so, estimated in January 2014 at 12 % [48].

ClinPak is a US-developed, Nigeria-implemented mobile EMR system designed to track a patient’s medical history, active medical problems and associated treatments in a point-of-care platform. It has been successfully implemented in Nigeria for improving maternal health, but found a new use during the EVD outbreak. Important especially early in the outbreak was information dissemination; ClinPak supported the development of other mobile apps to help disseminate EVD information [49].

Potential next steps that might be useful to streamline the use of EHRs and apps include:

1. Standardize the methodology in programming data in EHRs and apps;
2. Create and improve apps’ functionality;
3. Remove constraints on data input for contextualized diagnosis (e.g.*,* using the open.fda.gov model), and
4. Make information and usage available at point of care [50].

#### Discussion

According to the CDC, current estimates put the total number of cases at approximately 24,797 with about 8764 deaths since March 2014 [51, 52]. While the number of new cases has flattened out since the peak early summer of 2015, the crisis continues nearly two years after it began. Many fault the WHO for its mismanagement of the crisis during its earlier stages. Had there been a more concerted international effort at the onset of the crisis we may not have seen such a dramatic increase in the total number of cases. It is important to note that many of the cases seen outside of West Africa were the result of healthcare workers returning to their country of origin. Cybercare could have tracked all of the cases and allowed a more timely response to the disease outbreak.

With communicable diseases, we do not have the luxury to evacuate the patients in large numbers. We need to isolate diseased patients, treating them in place with either isolation (if they are infected) or quarantine (if they have had contact with infected individuals). Cybercare provides the electronic tools to allow this treatment to happen. The use of telemedicine and robotics is crucial to treating at a distance, allowing quarantine and isolation of individuals who are infected or exposed to the disease. If we transport these patients, we risk infecting the rest of the country or the world. This is what we began to see in Ebola in West Africa where individuals traveled out of the country with this highly deadly communicable disease.

In the future we need a healthcare system in place to treat pandemics when Ebola or Middle East Respiratory Syndrome coronavirus (MERS-CoV) infect individuals who travel internationally. The system will need to be able to stop the spread of disease with vaccines (i.e.*,* make vaccines rapidly with new technologies to produce large quantities in weeks rather than months; deliver vaccines with robotic-controlled drones); treat exposed or infected patients with isolation or quarantine; and track patients with both communicable disease as part of the pandemic, and non-communicable chronic diseases like diabetes that require ongoing treatment. The system should also *anticipate* a pandemic by examining susceptible populations, determining if any individuals are infected, and treating them early. Prior pandemics such as SARS had lower transmission and death rates than Ebola, whose mortality is extremely high.

Cybercare is ideal for remotely treating a pandemic because it provides telemedicine for treatment at a distance, along with aggressive task shifting, and the technology for advanced quarantine and isolation with robotics. As medical responders set up 11 hospitals in West Africa for Ebola, we could have positioned key technologies. An example: IVs were crucial in Ebola to reduce the fatality from 80 to 40 %. Yet, placing an IV in an Ebola patient is very dangerous for a pandemic provider (personal quote, Tom Crabtree). We could have placed explosive ordnance robots at those hospitals as remote-controlled nurses. Robot nurses already exist [53]. We need to teach the robots to place intravenous lines and care for patients in situations where the risk of provider infection is so high — this will certainly be possible before 2030.

Travel is very dangerous in a pandemic of Ebola or even SARS. Patients need to know and believe that by staying in place they will receive the best care possible. This will protect those across borders from becoming infected. In 2030, our response to pandemics will dramatically improve with Cybercare.

## Conclusion: the way forward

Cybercare will provide the foundation for healthcare delivery in the future. It is based on seven pillars of information technology (genomics; telemedicine; robotics; simulation, including virtual and augmented reality; AI; the EMR; and smartphones) that support three key paradigms. We will shift care from treatment to prevention, from specialist to generalist, and from the hospital to the home. Cybercare could help enhance private health and public health; address the GBD with treatment for communicable illnesses; and help the aging population cope with their chronic illnesses in the developed world.

Cybercare is already accomplishing many of the goals we outlined almost a decade ago [3]. Medical providers are available in some drugstores (we envisioned this in 2008, and it is now a reality), and via telemedicine (this was in early stages in 2008, and is now widely implemented). Many patient-monitoring devices and cell phone apps exist to collect health data for the use of both patients and providers.

Over the next 15 years, we will see a dramatic acceleration in the use of technology in health care. By 2030, we expect that much of what we have predicted in this paper will be in place in the US healthcare system and in the Global healthcare environment. Telemedicine is the oldest, best-known Cybercare technology, but that is rapidly changing as technologies evolve and merge. For example, telemedicine is now being done on a smartphone. New technologies will develop that enhance this model. Information fusion and techniques for management (of big data, information, knowledge and wisdom) promise to play a central future role in the prevention and detection of the burden of disease as well as its remediation. Oracle has implemented this technology in Indonesia, Singapore, and Australia [26].

What is key in the future (see Fig. 3) is that we can no longer concentrate on the individual. We need to understand the individual human being in four dimensions: physical, behavioral, genomics, and Internet — and how these parts interplay. We need to provide enough bandwidth as a resource to each individual to allow us to track them and how they fit into their macrobiome and microbiome. The Internet is the core of Cybercare’s functioning, and the Internet will need to be well protected during a crisis like Katrina or EVD.

The global burden of disease is the responsibility to which Cybercare must respond, whether for the individual patient or for an entire population. This is a tall order for any healthcare system, but a necessity if we are to be successful in reducing costs and simultaneously increasing quality. The Lancet Commission has forecasted a global shortage of surgeons in 2030 [35] unless we begin to shift our policy. This potential shortage can be addressed with task shifting and Cybercare technologies through simulation, teleconsultation, telerobotics, and telementoring using augmented reality.

This model and all these future plans are possible through new innovative technology — that some see as disruptive, but we see as a necessary next step — as we move forward to address the needs of our health care for individuals, our countries, and our world. As Cybercare evolves, the functions of private health care, public health care, and security will become more integrated. We need a dual-use system that can respond equally to communicable and non-communicable disease, and also to trauma, including acts of terror and war. Just as Eisenhower built the US national highway system, a massive and expensive project, by federal mandate — we see Cybercare II as a national healthcare security system that needs to be funded and put into place as soon as possible to protect us from communicable disease, non-communicable disease, trauma, and war. Similar to the US national highway system, Cybercare would be a dual-use system, capable of responding to daily concerns, and if needed to respond to crises whether natural or man-made. Other governments could join and follow with mandates of their own. This would allow us to sustain our societal goals of freedom and the healthy pursuit of happiness. Health is a cornerstone of our way of life.

Moving health care provision from a central, hospital-based model to a networked, distributed model (Cybercare) will improve outcomes and efficiency, facilitate access, improve security, and increase patient and provider satisfaction. This model will yield a strong and bright future for health care and will enable improved health quality and lower costs for all the citizens of the world.
